# Supplementary material for: Highly synergistic combinations of nanobodies that target SARS-CoV-2 and are resistant to escape
Source: eLife. 2021 Dec 15;10:e73027. doi: 10.7554/eLife.73027 (PMC8651292; doi:10.7554/eLife.73027)
Supplement: Figure 6—source data 1. [file elife-73027-fig6-data1.zip › receptor_nanobody_co-complexes/modeling_details.docx]

| **PDB file name** | **Receptor name (spike domain) and chain/(s)** | **Nanobody**  **name and chain** | **Crosslink satisfaction** | **Minimum distance between CDR3 and escape mutant** |
| --- | --- | --- | --- | --- |
| S1-RBD-9.pdb | RBD, chain “0” | S1-RBD-9, chain “A” | 3 out of 5 | **K378**: 7.80 Å |
| S1-RBD-15.pdb | RBD, chain “0” | S1-RBD-15, chain “A” | 7 out of 7 | **Y508:** 11.45 Å |
| S1-RBD-16.pdb | RBD, chain “0” | S1-RBD-16, chain “A” | N/A | **N354**: 5.75 Å |
| S1-RBD-21.pdb | RBD, chain “0” | S1-RBD-21, chain “A” | N/A | **F486**: 6.63 Å  **Y489**: 6.33 Å |
| S1-RBD-22.pdb | RBD, chain “0” | S1-RBD-22, chain “A” | N/A | **K378**: 5.76 Å |
| S1-RBD-23.pdb | RBD, chain “0” | S1-RBD-23, chain “A” | N/A | **S349**: 6.14 Å |
| S1-RBD-24.pdb | RBD, chain “0” | S1-RBD-24, chain “A” | N/A | **K378**: 6.76 Å  **P384**: 6.52 Å |
| S1-RBD-29.pdb | RBD, chain “0” | S1-RBD-29, chain “A” | N/A | **E484**: 6.03 Å |
| S1-RBD-35.pdb | RBD, chain “0” | S1-RBD-35, chain “A” | N/A | **T478**: 8.36 Å  **F486**: 6.21 Å  **Y489**: 9.13 Å |
| S1-RBD-40.pdb | RBD, chain “0” | S1-RBD-40, chain “A” | N/A | **F490**: 6.58 Å |
| S1-1.pdb | RBD, chain “0” | S1-1, chain “A” | 8 out of 12 | **Y369**: 6.82 Å  **G404**: 5.27 Å |
| S1-6.pdb | RBD, chain “0” | S1-6, chain “A” | N/A | **S371**: 6.08 Å |
| S1-23.pdb | RBD, chain “0” | S1-23, chain “A” | 7 out of 7 | **E484**: 6.17 Å  **F490**: 5.62 Å  **Q493**: 6.19 Å |
| S1-36.pdb | RBD, chain “0” | S1-36, chain “A” | N/A | **L452**: 6.79 Å  **F490**: 7.69 Å |
| S1-37.pdb | RBD, chain “0” | S1-37, chain “A” | N/A | **F490**: 7.47 Å |
| S1-46.pdb | RBD, chain “0” | S1-46, chain “A” | 4 out of 4 | N/A |
| S1-48.pdb | RBD, chain “0” | S1-48, chain “A” | N/A | **Y449**: 6.31 Å  **F490**: 7.53 Å  **S494**: 6.14 Å |
| S1-49.pdb | NTD, chain “0” | S1-49, chain “A” | 8 out of 8 | **S172**: 7.70 Å |
| S1-62.pdb | RBD, chain “0” | S1-62, chain “A” | N/A | **E484**: 6.21 Å |
| S2-10.pdb | S2, chains “0”, “1”, “2” | S2-10, chain “A” | 4 out of 4 | **S982**: 6.69 Å |
| S2-40.pdb | S2, chains “0”, “1”, “2” | S2-40, chain “A | 3 out of 3 | N/A |

N/A: no data available.

RBD: receptor binding domain on the S1 region of spike. Structure corresponds to residues 333-526 of 6m0j.E

NTD: N-terminal domain on the S1 region of spike. Structure corresponds to residues 16-305 of 7ly3.A

S2: Spike ectodomain. Structure corresponds to residues 689-1162 of each of the three chains 6xr8.A, 6xr8.B, 6xr8.C.

Min. distance between escape mutant on the receptor and CDR loops on the nanobody was restrained to be within 8 Å.
